# The impact of recreational cannabis legalization on pediatric emergency department visits in British Columbia, Canada

**DOI:** 10.24095/hpcdp.46.4.04

**Published:** 2026-04

**Authors:** Melody Xiao, Aygun Ibrahimova, Atousa Zargaran, Phoebe Cheng, Mojgan Karbakhsh, Fahra Rajabali, Kate Turcotte, Kirvy Quiambao, Shelina Babul

**Affiliations:** 1 BC Injury Research and Prevention Unit, British Columbia Children’s Hospital, Vancouver, British Columbia, Canada; 2 Canadian Hospitals Injury Reporting and Prevention Program, British Columbia Children’s Hospital, Vancouver, British Columbia, Canada; 3 Fraser Health Authority, Surrey, British Columbia, Canada; 4 British Columbia Children’s Hospital Research Institute, Vancouver, British Columbia, Canada; 5 Department of Pediatrics, The University of British Columbia, Vancouver, British Columbia, Canada

**Keywords:** child, adolescent, cannabis, edibles, poisoning, risk, legislation

## Abstract

**Introduction:**

Canadian youth report some of the highest rates of cannabis use globally, raising concerns about child and youth poisonings from unintentional exposures and recreational use following legalization. This study examines and compares trends in cannabis poisonings among children and youth aged 16 years or younger treated in the emergency department (ED) of a Canadian pediatric hospital before and after the legalization of non-medical cannabis.

**Methods:**

Cannabis-related ED visits at BC Children’s Hospital (BCCH) (2016–2021) were identified from the Canadian Hospitals Injury Reporting and Prevention Program (CHIRPP) database using injury codes and keyword searches. Key variables included age, sex, intent, method of cannabis use, poisoning intent, season, peer use, and mode of ED arrival. Chi-square tests were used to assess associations between characteristics, and interrupted time series analysis evaluated legalization impact.

**Results:**

There were 304 pediatric-related cannabis poisoning ED visits to BCCH between 2016 and 2021, increasing 55.5% from pre-legalization (n=119) to post-legalization (n=185). Unintentional poisonings rose from 4% to 12%, mainly involving the ingestion of edibles among children with a median age of 3 years. Ninety percent of cases involved intentional use, with co-consumption with other substances more common than cannabis use only. Interrupted time series analysis showed an upward trend in poisonings from 2016 to 2018, an immediate 48% increase in ED visits following legalization, followed by a decline.

**Conclusion:**

Findings highlight the need for strengthened substance use prevention efforts, education programs, and continued surveillance to reduce harm among children and youth from intentional use and unintentional cannabis exposures, particularly involving edibles.

**Highlights**

- ED visits at BC Children’s Hospital related to unintentional cannabis consumption primarily affected children with a median age of 3 years, increasing from 4% pre-cannabis legalization to 12% post-legalization.
- ED visits at BC Children’s Hospital related to poisoning from intentional cannabis use mostly involved individuals with an average age of 15 years, with co-consumption with other substances more common than cannabis-only both pre- and postcannabis legislation.
- After legalization, youth co-consumption poisonings seen at BC Children’s Hospital ED were more common among females, and youth cannabis-only poisonings increased on weekends.
- ED visits at BC Children’s Hospital for cannabis poisoning rose from 2016 to 2018, spiked 48% at legalization, and declined in the post-cannabis legalization period.

## Introduction

Canadian youth have some of the highest cannabis use rates worldwide, with 43% of youth aged 16 to 19 years reporting using cannabis at least once in the past 12 months as of 2023.^1^,^2^ The enactment of Bill C-45 (*The Cannabis Act*) on 17 October 2018, legalized and regulated the production, distribution, sale and possession of cannabis in Canada.^3^ While legalization was intended only for adults 18 years of age and older, it raised concern for increased intentional use as a result of increased access among adolescents and inadvertent exposure among young children, including unintentional poisonings.^4^ Unintentional cases usually involve young children inadvertently ingesting cannabis products, especially edibles, whereas intentional cases are more common in adolescents and linked to recreational use, overconsumption, polysubstance use, or self-harm. Despite the high prevalence of cannabis use among Canadian youth prior to legalization, concerns remain that health risks may be escalating, as reflected by increases in emergency department (ED) visits and changes in presentation patterns.^5^,^6^ These increases may result from increased social acceptability and shifts in perceptions, access, and consumption patterns among youth following cannabis legalization.^7^-^9^

In several US states with legalized recreational cannabis, intentional exposures among youth have risen (ages 12–24 years), reflected in increased ED visits, hospitalizations and poison control calls.^10^-^13^ In Massachusetts, over half of intentional exposure calls among adolescents aged 15 to 19 years involved polysubstance use, most often alcohol.^10^ Similar patterns appear in Canada, where adolescent cannabis-related ED visits and poison control centre calls increased following legalization, with most cases involving recreational use, many involving edibles, and self-harm exposures often involving multiple substances (ages 11–18).^14^-^17^ In the Canadian provinces of Alberta and Ontario, ED cases rose after recreational cannabis legalization, with a 20% increase in youth poisonings, particularly among adolescents aged 12 to 17 years.^14^ There are also notable changes in consumption patterns among youth in British Columbia (BC). The 2023 BC Adolescent Health Survey by the McCreary Centre Society showed a shift in cannabis consumption patterns, where compared to 2018, youth aged 12 to 18 were less likely to have smoked cannabis and more likely to have consumed it in edible form during their most recent use.^16^

Cannabis poses particular risks for children, who are susceptible to severe intoxication and long-term impacts on brain development.^18^ US studies show rising pediatric poisonings after cannabis legalization (ages 0–12 years), mostly from young children unintentionally ingesting edibles.^19^-^24^ Specifically in California, cannabis exposures rose sharply after legalization in 2016 and retail sales in 2018, with the largest increase in poison control calls among children under the age of 13 years and especially under the age of 6 years, driven by ingestion of cannabis gummies and candies.^25^ Similar patterns have emerged in Canada, where edibles, including food and drink products infused with cannabis, were legalized in 2019.^26^ Since then, unintentional pediatric exposures (ages 0–12 years), particularly from edibles, have risen, contributing to higher ED visits, hospitalizations and poison control calls across several provinces.^5^,^15^,^27^-^29^ Data from the British Columbia Drug and Poison Information Centre (BC DPIC), which managed nearly 4000 cannabis-related calls between 2013 and 2021, show steady increases in calls even before legalization, with the highest rates in calls for unintentional edible exposures among children under the age of 5.^15^ Another Canadian study found that among children aged 0 to 9 years, cannabis poisoning hospitalizations increased after dried cannabis flower legalization in 2018 and doubled after edibles legalization, where hospitalizations rose more in provinces permitting edibles (Ontario, Alberta, BC) compared to Quebec, a jurisdiction that prohibited edibles at that time.^30^ As of 2025, all Canadian provinces and territories allow edible cannabis sales, with variations in rollout timelines; however, Quebec currently restricts products that may appeal to minors, such as sweets, chocolates and desserts, permitting only select edibles such as dried fruits and nuts.^31^

There was also a notable rise in substance use among children and youth in the USA and Canada during the COVID-19 pandemic, coinciding with increased poisoning-related ED visits compared to pre-pandemic levels, with cannabis accounting for a growing proportion of poisonings. Relative increases were observed among young children (ages 6 months to 5 years) and adolescents (aged 11–18 years), with elevated proportions persisting into young adulthood (up to 24 years).^13^,^17^,^32^ One US study suggested that the increase in unintentional exposures among children aged 6 months to 5 years was likely linked to more time at home and greater availability of cannabis products in households.^32^ As for youth aged 11 to 24, the increased ED visits, particularly for recreational use and intentional self-harm, may be attributed to pandemic-related stressors and disruptions to routines.^13^,^17^

Understanding the impact of legislative changes on cannabis poisonings can guide prevention policies, given young children’s susceptibility to unintentional exposures and adolescents’ risk of intentional, often polysubstance, use, which may harm their health.^33^,^34^ A 2020 study examined cannabis poisonings in children and youth 16 years of age and younger seen at BC Children's Hospital (BCCH) before recreational cannabis legalization to establish a baseline.^35^ The purpose of this study is to examine trends and patterns in cannabis poisonings among children and youth under 16 years of age, seen at BCCH following the 2018 legalization of cannabis for non-medical purposes in Canada, and includes factors such as co-consumption, routes of exposure, and product types. Analyzing these changes can identify emerging risks, evaluate the effectiveness of existing safeguards like packaging and product labelling requirements, and inform development of targeted education for caregivers and communities.

Ethics approval was obtained from The University of British Columbia (UBC), Children’s and Women’s Health Centre of British Columbia (CW), Research Ethics Board; certificate number H23-00948.

## Methods

***Data collection and extraction***

Data on cannabis poisoning-related ED visits at BCCH from 1 January 2016 to 31 December 2021 were extracted from the Canadian Hospitals Injury Reporting and Prevention Program (CHIRPP) database. CHIRPP is an ED surveillance system that records injury cases, including poisonings, through the completion of forms collected at registration by patients or their caregivers. Cannabis-related poisoning cases were identified using CHIRPP codes “50NI” (poisoning or toxic effect) and “900BP” (body part not required), along with a keyword search in the injury descriptions for terms such as “cannabis,” “hash,” “CBD,” “marijuana,” “weed,” “THC,” “bong,” or “edible.” To ensure completeness, injury descriptions were reviewed to capture any missed cases. 

***Description of variables***

Key variables extracted included age, sex, intent of cannabis consumption, intent of poisoning, patient disposition, time of cannabis use, season, method of cannabis use, peer substance use, treatment-seeking individual, and mode of ED arrival.

Cannabis use was classified as unintentional (e.g. young children inadvertently ingesting edibles) or intentional (deliberate use for psychoactive effects). The intent of poisoning was classified as intentional (including but is not exclusive to self-harm or harm by others) or unintentional. Time of poisoning was categorized by time of day—morning (12:00 a.m.–11:59 a.m.), afternoon (12:00 p.m.–5:59 p.m.), and evening (6:00 p.m.–11:59 p.m.)—and by day of the week, grouped into weekdays (Monday to Friday) and weekends (Saturday and Sunday). Seasons were defined as spring (March–May), summer (June–August), autumn (September–November), and winter (December–February). Method of cannabis use included inhalation (e.g. joint, bong, vaporizer), ingestion (e.g. brownies, gummies), or both. Peer substance use indicated whether substances were used with peers. The treatment-seeking individual was the person who brought the patient to the ED—categorized as a bystander (not involved and unrelated), family or friend, or the patient themself. Mode of ED arrival included emergency health services (e.g. ambulance or police), family, or other (e.g. self-admittance, social worker, or friend).

***Data analyses***

Chi-square tests were used to assess whether the distribution of cannabis-only and co-consumption poisoning cases differed across categorical variables such as sex and timing. An interrupted time series (ITS) analysis using segmented Poisson regression evaluated the immediate and long-term impact of cannabis legalization on cannabis poisoning-related ED visits. Due to the low number of monthly cases, data were aggregated quarterly, resulting in only 24 data points available for analysis. Given the small number of observations, the study was underpowered to detect modest effect, and significance was assessed at the 10% level to reduce the risk of false negatives.

The post-legalization period was defined as starting on 1 January 2019 to account for the gradual rollout and limited initial supply from licensed cannabis producers.^36^ The study period was divided into pre-legalization (Q1 2016–Q4 2018) and post-legalization (Q1 2019–Q4 2021). The outcome variable was the number of cannabis poisoning cases, and the exposure variable was cannabis legalization, marked in Q1 2019. To capture the multiplicative effect of quarterly changes, the ITS model coefficient was exponentiated to produce a rate ratio. All the data analyses were conducted using R, version 4.2.3.^37^

## Results

During the 3-year pre-legalization period (January 2016–December 2018), there were 119 ED visits for pediatric cannabis poisoning at BCCH.^35^ After legalization (January 2019–December 2021), this rose to 185 cases, representing a 55.5% increase. In both periods, over 85% of cases involved intentional cannabis use, with few resulting from unintentional consumption.

***Unintentional cannabis consumption***

After cannabis legalization, 12% of cannabis-related poisonings at BCCH involved unintentional consumption, compared to 4% pre-legalization.^35^ In both periods, the median age was 3 years, with most cases involving the ingestion of edibles like cookies, gummies or chocolate. Most patients were treated and discharged from the ED, with few requiring admissions. 

Before legalization, 80% of ED visits related to unintentional cannabis consumption involved males, with 73% on weekends.^35^ After legalization, 59% involved females, with most on weekdays. In the post-legalization period, 90% of cases occurred after edibles were legalized in late 2019.

***Intentional cannabis consumption***

Intentional cannabis use was classified as cannabis-only or co-consumption with alcohol, illicit drugs, or medications. It accounted for 96% of cases pre-legalization and 88% post-legalization. Among 114 pre-legalization cases, 29% involved cannabis only and 71% co-consumption.^35^ Post-legalization, 34% of the 163 patients used cannabis only, and 66% reported co-consumption. 

The demographic comparison of intentional cannabis poisoning at BCCH before and after legalization is presented in Table 1. The median patient age was 15 years (IQR:14–16 years) for both periods. Pre-legalization, cannabis-only and co-consumption cases were evenly distributed by sex (*p*=0.29).^35^ Post-legalization, male cases were more likely to be cannabis-only use, while co-consumption was more common among females (*p*=0.01). For co-consumption specifically, males were more likely to co-consume before legalization, while females were more likely to co-consume afterward (*p*<0.01). Over 85% of cannabis poisonings were due to recreational use rather than self-harm or assault. Nearly 90% of patients were treated or observed in the ED and discharged during both periods. 

**Table 1:** Demographics of patients seen at the emergency department of British Columbia Children’s Hospital due to poisoning resulting from the intentional consumption of cannabis, pre- and post-legalization, CHIRPP, January 2016 to December 2021

Descriptive	Substance used					
	Pre-legalization January 2016–December 2018 (n = 114)			Post-legalization January 2019–December 2021 (n = 163)		
	Cannabis co-consumption		Chi-square χ^2^, *p*, *df*	Cannabis co-consumption		Chi-square χ^2^, *p*, *df*
	No n (%)	Yes n (%)		No n (%)	Yes n (%)	
Median age in years (IQR)	15 (14–15)	15 (14–16)	N/A	15 (14–15)	15 (14–16)	N/A
Sex						
Male	16 (49%)	48 (59%)	χ^2^ = 1.11, *p* = 0.29, *df* = 1	33 (60%)	39 (36%)	χ^2^ = 7.49, *p* = 0.01, *df* = 1
Female	17 (52%)	33 (41%)	χ^2^ = 1.11, *p* = 0.29, *df* = 1	22 (40%)	69 (64%)	χ^2^ = 7.49, *p* = 0.01, *df* = 1
Intent of poisoning						
Unintentional	45 (98%)	59 (87%)	^b^	53 (96%)	87 (81%)	^b^
Intentional self-harm	^a^	6 (9%)	^b^	^a^	12 (11%)	^b^
Other intents	^a^	^a^	^b^	^a^	8 (7%)	^b^
Patient disposition						
No treatment (advice only, diagnostic testing, referred to GP)	7 (21%)	19 (24%)	^b^	16 (29%)	21 (19%)	^b^
Treated, follow-up may or may not be required	7 (21%)	27 (33%)	^b^	17 (36%)	35 (32%)	^b^
Observation, follow-up may or may not be required	16 (49%)	26 (32%)	^b^	12 (22%)	31 (29%)	^b^
Admittance into hospital for treatment	^a^	8 (10%)	^b^	5 (11%)	13 (12%)	^b^
Other treatments	^a^	^a^	^b^	^a^	8 (7%)	^b^

**Abbreviations: **CHIRPP, Canadian Hospitals Injury Reporting and Prevention Program; *df*, degrees of freedom; GP, general practitioner; IQR, interquartile range. 

**Notes: **Pre-legalization table adapted from Table 1 in Cheng P et al.^35^ Adapted with permission. 

"Other intents" are unspecified assault or event of undetermined intent. 

Other treatments are admitted primarily for a reason other than poisoning treatment. 

^a^ Absolute frequencies of fewer than five. 

^b^ Absence of a χ^2^ test due to the violation of one or more assumptions of the test. 

During the pre-legalization period, cannabis-only and co-consumption poisoning differed significantly in timing (Table 2).^35^ Nearly half of cannabis-only cases occurred in the afternoon, with the rest occurring in the evening (*p*< 0.01).^35^ Co-consumption cases occurred mostly in the evening (58%), followed by morning (24%) and afternoon (16%) (*p* = 0.01).^35^ Post-legalization, time of day was unknown for 33% of cannabis-only cases and 58% of co-consumption cases. Poisonings were more common on weekdays pre-legalization (*p*=0.01)^35^ with a rise in weekend cannabis-only poisonings observed post-legalization. Seasonality was associated with poisoning type pre-legalization (*p*=0.05), with fewer cannabis-only cases in spring (15%) and fewer co-consumption cases in winter (12%).^35^ No seasonal association was found post-legalization. 

**Table 2:** Temporal distribution of cannabis and co-consumption poisoning due to intentional consumptions seen at the emergency department of British Columbia Children’s Hospital, pre- and post-legalization, CHIRPP, January 2016 to December 2021

Descriptive	Substance used					
	Pre-legalization January 2016–December 2018 (n = 114)			Post-legalization January 2019–December 2021 (n = 163)		
	Cannabis co-consumption		Chi-square χ^2^, *p*, *df*	Cannabis co-consumption		Chi-square χ^2^, *p*, *df*
	No n (%)	Yes n (%)		No n (%)	Yes n (%)	
Time of the day						
Morning	^a^	19 (24%)	**χ^2^ = 11.86, *p* < 0.01, *df* = 2^d^**	7 (13%)	12 (11%)	^b^
Afternoon	15 (46%)	13 (16%)	**χ^2^ = 11.86, *p* < 0.01, *df* = 2^d^**	18 (33%)	17 (16%)	^b^
Evening	14 (42%)	47 (58%)	**χ^2^ = 11.86, *p* < 0.01, *df* = 2^d^**	12 (22%)	16 (15%)	^b^
Unknown	^a^	^a^	**χ^2^ = 11.86, *p* < 0.01, *df* = 2^d^**	18 (33%)	63 (58%)	^b^
Time of the week						
Weekday	30 (91%)	56 (69%)	**χ^2^ = 6.00, *p* = 0.01, *df* = 1^d^**	39 (71%)	70 (65%)	χ^2^ = 0.37, *p* = 0.54, *df* = 1
Weekend	^a^	25 (31%)	**χ^2^ = 6.00, *p* = 0.01, *df* = 1^d^**	16 (29%)	38 (35%)	χ^2^ = 0.37, *p* = 0.54, *df* = 1
Season						
Spring	5 (15%)	23 (28%)	χ^2^ = 7.76, *p* = 0.05, *df* = 3	9 (16%)	17 (16%)	χ^2^ = 5.39, *p* = 0.15, *df* = 3
Summer	10 (30%)	18 (22%)	χ^2^ = 7.76, *p* = 0.05, *df* = 3	20 (36%)	26 (24%)	χ^2^ = 5.39, *p* = 0.15, *df* = 3
Autumn	8 (24%)	30 (37%)	χ^2^ = 7.76, *p* = 0.05, *df* = 3	18 (33%)	33 (31%)	χ^2^ = 5.39, *p* = 0.15, *df* = 3
Winter	10 (30%)	10 (12%)	χ^2^ = 7.76, *p* = 0.05, *df* = 3	8 (15%)	32 (30%)	χ^2^ = 5.39, *p* = 0.15, *df* = 3
Year						
2016	8 (24%)	27 (33%)	χ^2^ = 1.2, *p* = 0.55, *df* = 2	^c^	^c^	^c^
2017	10 (30%)	25 (31%)	χ^2^ = 1.2, *p* = 0.55, *df* = 2	^c^	^c^	^c^
2018	15 (46%)	29 (36%)	χ^2^ = 1.2, *p* = 0.55, *df* = 2	^c^	^c^	^c^
2019	^c^	^c^	^c^	23 (42%)	49 (45%)	χ^2^ = 2.11, *p* = 0.35, *df* = 2
2020	^c^	^c^	^c^	20 (36%)	28 (26%)	χ^2^ = 2.11, *p* = 0.35, *df* = 2
2021	^c^	^c^	^c^	12 (22%)	31 (29%)	χ^2^ = 2.11, *p* = 0.35, *df* = 2

**Abbreviations:** CHIRPP, Canadian Hospitals Injury Reporting and Prevention Program; *df*, degrees of freedom. 

**Notes:** Pre-legalization table adapted from Table 2 in Cheng P et al.^35^ Adapted with permission. 

Bolded values indicate significant findings at the *p* < 0.05 level. 

^a^ Absolute frequencies of fewer than five. 

^b^ Due to a large number of unknown cases distorting the analysis, the chi-squared results are not displayed. 

^c^ Absence of a χ^2^ test due to the violation of one or more assumptions of the test. 

Inhalation alone was the most common method of cannabis consumption for both cannabis-only and co-consumption poisonings (Table 3). Over half of cannabis poisoning cases involved peer substance use for both cannabis-only and co-consumption for both periods. Pre-legalization, individuals who used only cannabis were most often brought in by family members, and those who co-consumed cannabis with other substances were more often brought in by both bystanders and family (*p*=0.01). Post-legalization, family continued to be the main group helping individuals access treatment, for both cannabis-only use and co-use with alcohol. Emergency health services (EHS), including police and ambulance (ground or air), accounted for most ED arrivals for both cannabis-only and co-consumption poisonings during both the pre- and post-legalization periods.

**Table 3:** Characteristics of cannabis and co-consumption poisonings from intentional consumptions seen at the emergency department of British Columbia Children’s Hospital, pre- and post-legalization, CHIRPP, January 2016 to December 2021

Descriptive	Substance used					
	Pre-legalization January 2016–December 2018 (n = 114)			Post-legalization January 2019–December 2021 (n = 163)		
	Cannabis co-consumption		Chi-square χ^2^, *p*, *df*	Cannabis co-consumption		Chi-square χ^2^, *p*, *df*
	No n (%)	Yes n (%)		No n (%)	Yes n (%)	
Method of cannabis use						
Inhalation	20 (67%)	59 (81%)	^b^	27 (53%)	56 (55%)	^b^
Ingestion	10 (33%)	^a^	^b^	14 (27%)	14 (14%)	^b^
Multiple	^a^	^a^	^b^	^a^	^a^	^b^
Unknown	^a^	10 (14%)	^b^	6 (12%)	30 (29%)	^b^
Peer substance use						
No	11 (33%)	13 (16%)	χ^2^ = 2.93, *p* = 0.09, *df* = 1	8 (15%)	9 (8%)	^c^
Yes	18 (55%)	49 (61%)	χ^2^ = 2.93, *p* = 0.09, *df* = 1	18 (33%)	58 (54%)	^c^
Unknown	^a^	19 (24%)	χ^2^ = 2.93, *p* = 0.09, *df* = 1	29 (53%)	41 (38%)	^c^
Treatment-seeking individual						
Bystander	^a^	32 (40%)	**χ^2^ = 9.14, *p* = 0.01, *df* = 2^d^**	13 (24%)	28 (26%)	^c^
Patient	8 (24%)	9 (11%)	**χ^2^ = 9.14, *p* = 0.01, *df* = 2^d^**	5 (9%)	10 (9%)	^c^
Family	15 (45%)	28 (35%)	**χ^2^ = 9.14, *p* = 0.01, *df* = 2^d^**	16 (29%)	43 (40%)	^c^
Unknown	6 (18%)	9 (15%)	**χ^2^ = 9.14, *p* = 0.01, *df* = 2^d^**	21 (38%)	27 (25%)	^c^
Mode of ED arrival						
EHS	23 (70%)	72 (89%)	^b^	28 (51%)	82 (76%)	^b^
Family	7 (21%)	5 (6%)	^b^	9 (16%)	10 (9%)	^b^
Other(s)	^a^	^a^	^b^	^a^	^a^	^b^
Unknown	^a^	^a^	^b^	17 (31%)	15 (14%)	^b^

**Abbreviations:** CHIRPP, Canadian Hospitals Injury Reporting and Prevention Program; *df*, degrees of freedom; ED, emergency department; EHS, emergency health services. 

**Notes: **Pre-legalization table adapted from Table 3 in Cheng P et al.^35^ Adapted with permission. 

Bolded values indicate significant findings at the *p* < 0.05 level. 

^a^ Absolute frequencies of fewer than five. 

^b^ Absence of a χ^2^ test due to the violation of one or more assumptions of the test. 

^c^ Due to a large number of unknown cases distorting the analysis, the chi-squared results are not displayed. 

***Impact of cannabis legalization***

**Figure 1:**
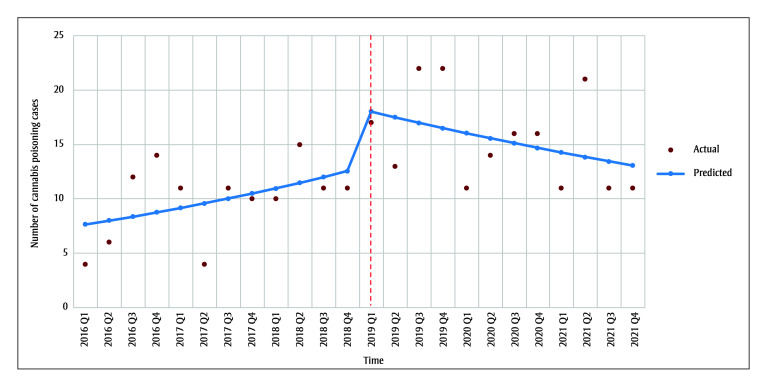
Interrupted time series analysis of cannabis poisonings seen at the emergency department of British Columbia Children’s Hospital, pre- and post-legalization, CHIRPP, January 2016 to December 2021

**Abbreviation:** CHIRPP, Canadian Hospitals Injury Reporting and Prevention Program. 

**Note: **The red dotted line represents the timing of cannabis legalization as defined in this study. 

The observed and predicted trends in child cannabis poisoning cases are shown in Figure 1, highlighting the impact of the cannabis legislation, with the interrupted time series results provided in Table 4. From 2016 to 2021, there was a non-significant average increase per quarter of 4.6% in the number of BCCH ED cannabis poisoning cases. Cannabis legalization was associated with an immediate increase of 47.9% (90% confidence interval [CI]: 3.0%–113.1%) in cannabis poisoning cases. This increase was statistically significant at the 10% level (*p*=0.076). Since the 2019 legalization, cannabis poisoning cases have declined by 7.1% per quarter (90%CI:−12.3%, −1.8%, *p*=0.030), a decrease that is statistically significant at the 5% level.

**Table 4:** Interrupted time series analysis of cannabis poisonings seen at the emergency department of British Columbia Children’s Hospital in relation to cannabis legalization, CHIRPP, January 2016 to December 2021

Predictor	% change (from rate ratio)	90% confidence interval	*p* value
**Pre-legalization trend**	4.6%	0.1%, 9.3%	0.093^*^
**Change at time of legalization**	47.9%	3.0%, 113.1%	0.076^*^
**Trend since legalization**	−7.1%	−12.3%, −1.8%	0.030^*^

**Abbreviation: **CHIRPP, Canadian Hospitals Injury Reporting and Prevention Program 

* Indicates statistical significance at the 10% level (*p* < 0.10). 

## Discussion

Our study showed that cannabis-related ED visits at BCCH rose yearly from 2016 to 2018, with an immediate 48% increase at legalization, after which the rate of increase declined. Data from the McCreary Centre Society showed that self-reported cannabis uses among youth aged 12 to 19 years fell from 25% in 2018 to 22% in 2023.^16^ BC DPIC reported that although cannabis poisoning calls rose from 2013 through 2021, cannabis legalization did not have an immediate effect on the rate of cannabis poisoning cases, and that the rate of increase slowed post-legalization.^15^

Building on these trends, unintentional cannabis poisonings remain a concern, particularly among young children. Our findings showed that during the post-legalization period, 90% of inadvertent cannabis consumption by young children occurred after the legalization of edibles in late 2019. This pattern extended beyond BC, as hospitalization data from Ontario and Alberta show a rise in unintentional cannabis poisonings among children aged 0 to 9 years with the increase in the commercial sale of cannabis edibles in 2019.^30^ BC DPIC reported that children under the age of 5 years consistently had the highest rates of unintentional cannabis edible exposures from 2013 to 2021. While poison centre calls involving edibles continued to rise after legalization, the pace of increase was slower than during the pre-legalization period.^15^ Despite this difference from our ED findings, the continued rise in pediatric exposures points to greater household availability of cannabis edibles post-legalization, increasing the risk of unintentional ingestion. Canada’s *Cannabis Act* requires all cannabis products to be sold in plain packaging and child-resistant containers, restricts promotions that could appeal to youth, mandates Health Warning Messages on packaging as well as display of the standardized cannabis symbol, and limits edible cannabis to a maximum of 10 mg THC per package to reduce the risk of unintentional overconsumption.^38^ Nevertheless, overconsumption may still occur if multiple packages are opened and stored together, illicit products are used or if products are made at home. These findings highlight the need for stronger poisoning prevention strategies, including public education on safe storage and awareness to help families distinguish legal from illegal products, as well as continued efforts to shift consumers toward the legal market.

Nearly 90% of pediatric cannabis-related ED visits in BC involved intentional use, mainly among youth with an average age of 15 years, with inhalation being the most common mode of consumption both before and after legalization. This ongoing predominance likely reflects, in part, the legalization of inhaled cannabis extracts in late 2019. Age-based differentiation in routes of exposure aligns with previous research—that ingestion is more common in children under 10 years of age while inhalation tends to be more prevalent among older youth, highlighting age-specific differences in access and modes of consumption.^10^,^11^,^21^ In Canada, intentional cannabis use among youth is frequently associated with the co-consumption of other substances, particularly alcohol.^35^ This pattern is not unique to Canada, as European surveys of youth aged 15 to 20 years report most adolescent cannabis use occurs with alcohol, and US surveys indicate that about 20% of Grade 12 students use alcohol and cannabis simultaneously.^39^,^40^ Teens who co-consume alcohol and cannabis tend to exhibit more problematic behavioural and substance use profiles; they smoke and drink more, and are more likely to experience social and behavioural challenges.^41^

Sex-based patterns in intentional consumption shifted after legalization. Before legalization, cannabis-only and co-consumption poisonings were similarly distributed between males and females. Post-legalization, females were more likely to present with co-consumption and accounted for a larger proportion of ED admissions. This may reflect the overlap with the COVID-19 pandemic, during which some evidence suggests that adolescent girls were more likely to use cannabis as a coping mechanism.^13^ Another possible factor is the increased social acceptability of cannabis following legalization, which may have particularly influenced female youth.^42^ The narrowing gender gap in cannabis use appears to be shaped by changing social and gender norms, as well as increased accessibility and normalization.

According to the McCreary Centre Society, youth most often cite experimentation, fun and peer influence as reasons for cannabis use, though some report using it to manage stress or pain.^16^ Strengthening family communication and school connectedness may help reduce risks, as a 2022 study found both factors had a direct protective effect against recent cannabis use among middle and high school students.^43^ School connectedness includes feeling cared for and participating in school activities; and supportive family practices include regular family meals, family routines, and strong parent-child relationships.^44^ A meta-analysis of youth cannabis prevention programs found that school-based prevention programs combining drug education with emotional and behavioural skill-building are more effective than those relying only on social influence (e.g. resisting peer pressure).^45^ School-based programs that last longer tend to be more effective at reducing cannabis use among youth. These programs produce stronger outcomes by making students less likely to start or more likely to reduce cannabis use, as longer duration allows more opportunities to reinforce prevention messages.^45^

Beyond school-based programs, strong regulations play a critical role in preventing youth cannabis use. In Canada and the USA, similar strict age limits apply, legalizing cannabis use for adults aged 18 years and older. Licensed retailers must verify the customer’s age for all purchases, often using scanners, making it difficult for underage individuals to purchase cannabis.^46^ For online purchases, customers are required to confirm their age at the point of sale, and upon delivery, couriers must verify the recipient’s age by checking government-issued identification to ensure compliance with legal requirements.^47^ Additionally, regulations limit the proximity of cannabis stores to sensitive areas; for instance, Ontario requires a minimum distance of 150 metres from schools, Alberta mandates 100 metres, and Edmonton has extended buffers to 200 metres from schools, parks and libraries.^48^ Because of these strict regulations, youth face barriers to accessing cannabis through legal channels and often turn to illegal markets, where no such restrictions exist.^49^ The McCreary Centre Society survey found that 10% of youth aged 12 to 18 years reported buying cannabis from a store, and 3% had bought it online.^16^ While the survey did not specify whether it was from the illegal market, the findings highlight the ongoing need to strengthen efforts to limit youth access to cannabis.

***Strengths and limitations***

Few studies have examined the impact of cannabis legalization in BC. This paper builds upon our prior work, which analyzed the landscape of pediatric cannabis poisonings at BCCH before legalization.^35^ By continuing surveillance and analyzing patient records following legalization, this study examines post-legalization records to show how policy changes affected pediatric cannabis-related ED visits.

The study period includes the COVID-19 pandemic, during which data collection practices were adapted to minimize virus transmission. To reduce contact, CHIRPP forms were not distributed to patients; incident details were abstracted from medical charts; staff contact with patients was limited, and discharges were expedited due to COVID-19 policies that restricted in-person interactions to reduce the risk of infection. Because of the overlap with the COVID-19 pandemic, it is not possible to disentangle the effects of cannabis legalization from pandemic-related shifts in health care use and youth-substance-use patterns. Although Canadian data showed a decline in youth substance-related ED visits during the early pandemic period, this may be attributed to changes in care-seeking behaviour, particularly if poisoning cases were milder and less likely to visit hospital.^50^ Therefore, ED visit data may be undercounted. Another limitation pertains to the ascertainment of intent, as limited interaction with patients during the pandemic made it more difficult to assess whether the poisoning was intentional or unintentional. Ongoing surveillance of pediatric ED visits in the post-legalization and post-pandemic periods is essential for tracking trends and patterns in cannabis-related harms. This will allow the public to evaluate the effectiveness of current regulations, and inform timely, evidence-based policy and prevention strategies.

## Conclusion

The number of pediatric-related cannabis ED visits seen at BCCH rose from 119 pre-legalization to 185 post-legalization; however, while visits were rising, the rate of increase declined following legalization. Most cannabis poisonings involved intentional inhalation, with a median age of 15 years, across both pre- and post-legalization periods. After legalization, females were more likely to present with co-consumption and accounted for a larger proportion of ED visits. This study highlights the characteristics of unintentional and intentional cannabis exposures among children and youth. These findings underscore the importance of ongoing surveillance, including the need to create positive social environments, promote school-based education programs, and increased enforcement to reduce youth access and minimize harm among children and youth. The post-legalization shifts in sex-based patterns, including rises in co-consumption among adolescent females, point to the need for gender-sensitive substance use prevention strategies. In addition, the rise in unintentional edible exposures among young children reinforces the need for strengthened poisoning prevention efforts, particularly targeting cannabis edibles.

## Acknowledgements

The authors would like to thank the Public Health Agency of Canada, the BCCH CHIRPP team members for data collection, and the BC Injury Research and Prevention Unit for their support and guidance. 

## Conflicts of interest

The authors declare that they have no conflicts of interest. 

## Authors’ contributions and statement

AZ, SB, PC conceptualized the plan and objectives for this study. AZ led the data collection. MX conducted the analyses and interpretation of the results, and drafted the manuscript. AI conducted the literature review, drafted the manuscript and contributed to interpreting results. FR, KQ, MK helped with the analysis and interpretation of the results. KT provided support with the ethics application and interpretation. All authors contributed to the review and revision of the manuscript. All authors read and approved the final manuscript. 

The content and views expressed in this article are those of the authors and do not necessarily reflect those of the Government of Canada.

## References

[B01] Health Canada releases new data on cannabis use in Canada [Internet]. Health Canada.

[B02] Charrier L, Dorsselaer S, Canale N, Baska T, Kilibarda B, Comoretto RI, et al A focus on adolescent substance use in Europe, central Asia and Canada: Health Behaviour in School-aged Children international report from the 2021/2022 survey, Volume 3 [Internet]. WHO Regional Office for Europe.

[B03] Cannabis legalization and regulation [Internet]. Justice Canada.

[B04] Karbakhsh M, Smith J, Pike I (2018). “Where does the high road lead?” Potential implications of cannabis legalization for pediatric injuries in Canada. Can J Public Health.

[B05] Varin M, Champagne A, Venugopal J, Li L, McFaull SR, Thompson W, et al (2023). Trends in cannabis-related emergency department visits and hospitalizations among children aged 0–11 years in Canada from 2015 to 2021: spotlight on cannabis edibles. BMC Public Health.

[B06] Weaver CG, Janz K, Haines-Saah R, Lang E (2020). Clearing the air: a study of cannabis related presentations to urban Alberta emergency departments following legalization. CJEM.

[B07] Fischer B, Russell C, Sabioni P, Brink W, Foll B, Hall W, et al (2017). Lower-Risk Cannabis Use Guidelines: a comprehensive update of evidence and recommendations. Am J Public Health.

[B08] Stone AL (2020). Adolescent cannabis use and perceived social norm trends pre-and post-implementation of Washington State’s liberalized recreational cannabis policy: Healthy Youth Survey, 2008–2018. Stone AL.

[B09] Gohari MR, Romano I, Leatherdale ST (2021). Changes in cannabis use modes among Canadian youth across recreational cannabis legalization: data from the COMPASS prospective cohort study. Addict Behav.

[B10] Whitehill JM, Harrington C, Lang CJ, Chary M, Bhutta WA, Burns MM, et al (2019). Incidence of pediatric cannabis exposure among children and teenagers aged 0 to 19 years before and after medical marijuana legalization in Massachusetts. JAMA Netw Open.

[B11] Wang GS, Davies SD, Halmo LS, Sass A, Mistry RD (2018). Impact of marijuana legalization in Colorado on adolescent emergency and urgent care visits. J Adolesc Health.

[B12] Harvey T, Gomez R, Wolk B, Ozcan A (2022). Varied presentations of pediatric patients with positive cannabinoid tests. Cureus.

[B13] Roehler DR, H IV, Radhakrishnan L, Holland KM, Gates AL, Vivolo-Kantor AM, et al (2023). Cannabis-involved emergency department visits among persons aged <25 years before and during the COVID-19 pandemic — United States, 2019–2022. Roehler DR, Smith H IV, Radhakrishnan L, Holland KM, Gates AL, Vivolo-Kantor AM, et al.

[B14] Callaghan RC, Sanches M, Heiden J (2023). Impact of Canada’s cannabis legalisation on youth emergency department visits for cannabis-related disorders and poisoning in Ontario and Alberta, 2015-2019. Callaghan RC, Sanches M, Vander Heiden J, Kish, S.

[B15] Trieu J, Dobbin N, Henderson SB, McVea D (2025). Impact of legalization on cannabis exposure calls to the British Columbia Poison Control Centre. Can J Public Health.

[B16] Smith A, Peled M, Poon C, Anderson L, Casey E (2025). Blunt Talk III: cannabis use among BC youth aged 12–18. McCreary Centre Society.

[B17] Davis A, Finkelstein Y, Rosenfield D (2022). The effects of COVID-19 on poisonings in the paediatric emergency department. Paediatr Child Health.

[B18] Stoner MJ, Dietrich A, Lam SH, Wall JJ, Sulton C, Rose E (2023). Marijuana use in children: an update focusing on pediatric tetrahydrocannabinol and cannabidiol use. Clinical Toxicology.

[B19] Thomas AA, Derau K, Bradford MC, Moser E, Garrard A, Mazor S (2019). Unintentional pediatric marijuana exposures prior to and after legalization and commercial availability of recreational marijuana in Washington State. J Emerg Med.

[B20] Thomas AA, Dickerson-Young T, Mazor S (2021). Unintentional pediatric marijuana exposures at a tertiary care children's hospital in Washington State: a retrospective review. Pediatr Emerg Care.

[B21] Dean D, Passalacqua KD, Oh SM, Aaron C, Harn MG, King A (2021). Pediatric cannabis single-substance exposures reported to the Michigan Poison Center from 2008–2019 after medical marijuana legalization. J Emerg Med.

[B22] Wang GS, Hoyte C, Roosevelt G, Heard K (2019). The continued impact of marijuana legalization on unintentional pediatric exposures in Colorado. Clin Pediatr.

[B23] Wang GS, Lait MC, Deakyne SJ, et al (2016). Unintentional pediatric exposures to marijuana in Colorado, 2009–2015. 2016;170(9):e160971.https://doi.org/10.

[B24] Wang GS, Roosevelt G, Lait MC, Deakyne SJ, Bronstein AC, Bajaj L, et al (2014). Association of unintentional pediatric exposures with decriminalization of marijuana in the United States. Ann Emerg Med.

[B25] Roth W, Tam M, Bi C, Kim J, Lewis J, et al (2022). Changes in California cannabis exposures following recreational legalization and the COVID-19 pandemic. Clin Toxicol.

[B26] Regulations under the Cannabis Act [Internet]. Health Canada.

[B27] Coret A, Rowan-Legg A (2022). Unintentional cannabis exposures in children pre-and post-legalization: a retrospective review from a Canadian paediatric hospital. Paediatr Child Health.

[B28] Myran DT, Cantor N, Finkelstein Y, Pugilese M, Guttman A, Jesseman R, et al (2022). Unintentional pediatric cannabis exposures after legalization of recreational cannabis in Canada. JAMA Netw Open.

[B29] Cohen N, Blanco L, Davis A, Kahane A, Mathew M, Schuh S, et al (2022). Pediatric cannabis intoxication trends in the pre and post-legalization era. Pediatric cannabis intoxication trends in the pre and post-legalization era. Clin Toxicol (Phila).

[B30] Myran DT, Tanuseputro P, Auger N, Konikoff L, Talarico R, Finkelstein Y (2023). Pediatric hospitalizations for unintentional cannabis poisonings and all-cause poisonings associated with edible cannabis product legalization and sales in Canada. JAMA Health Forum.

[B31] Rowe DJ Cannabis-infused poutine sauce, jerky, nuts among new edible options in Quebec [Internet]. Rowe DJ.

[B32] Laudone TW, Leonard JB, Hines EQ, Seung H (2022). Changes in unintentional cannabis exposures in children 6 months to 5 years reported to United States poison centers during the first nine months of the coronavirus-19 pandemic. Clin Toxicol.

[B33] Burggren AC, Shirazi A, Ginder N, London ED (2019). Cannabis effects on brain structure, function, and cognition: considerations for medical uses of cannabis and its derivatives. Am J Drug Alcohol Abuse.

[B34] Jacobus J, Tapert SF (2014). Effects of cannabis on the adolescent brain. Curr Pharm Des.

[B35] Cheng P, Zagaran A, Rajabali F, Turcotte K, Babul S (2020). Setting the baseline: a description of cannabis poisonings at a Canadian pediatric hospital prior to the legalization of recreational cannabis. Health Promot Chronic Dis Prev Can.

[B36] Cherney MA Canada’s struggle to supply legal weed described as ‘national shortage’ that could last months [Internet]. Cherney MA.

[B37] (2023). R: a language and environment for statistical computing. R Foundation for Statistical Computing.

[B38] Packaging and labelling guide for cannabis products. Health Canada.

[B39] Patrick ME, Veliz PT, Terry-McElrath YM (2017). High-intensity and simultaneous alcohol and marijuana use among high school seniors in the U.S. Subst Abus.

[B40] Pape H, Rossow I, Storvoll EE (2009). Under double influence: assessment of simultaneous alcohol and cannabis use in general youth populations. Drug Alcohol Depend.

[B41] Chun TH, Spirito A, ndez L, Fairlie AM, Sindelar-Manning H, Eaton CA, et al (2010). The significance of marijuana use among alcohol using adolescent ED patients. Acad Emerg Med.

[B42] Matheson J, Foll B (2023). Impacts of recreational cannabis legalization on use and harms: a narrative review of sex/gender differences. Impacts of recreational cannabis legalization on use and harms: a narrative review of sex/gender differences. Front Psy-chiatry.

[B43] Clements-Nolle KD, Lensch T, Drake CS, Pearson JL (2022). Adverse childhood experiences and past 30-day cannabis use among middle and high school students: the protective influence of families and schools. Addict Behav.

[B44] Preventing youth marijuana use: factors associated with use [Internet]. EDC.

[B45] Porath-Waller AJ, Beasley E, Beirness DJ (2010). A meta-analytic review of school-based prevention for cannabis use. Health Educ Behav.

[B46] Cannabis legalization: considerations for children and young people [Internet]. CCSA.

[B47] (2022). Buying cannabis – what you need to know [Internet]. Public Safety Canada.

[B48] The legalities of store location considerations [Internet]. Cannabis Retailer.

[B49] A public health perspective on cannabis legalization and regulation in Canada [Internet]. CCSA.

[B50] Dharma C, Al-Jaishi AA, Collins E, Orchard C, Amankwah N, Lang JJ, et al (2024). Assessing the impact of the COVID-19 pandemic on the mental health– related hospitalization rate of youth in Canada: an interrupted time series analysis. Health Promot Chronic Dis Prev Can.

